# Young adults with eating disorders perspectives on educational resources to support the transition into adult medicine: a thematic analysis

**DOI:** 10.1186/s40337-023-00771-6

**Published:** 2023-03-23

**Authors:** Jennifer Mooney, Anna Dominic, Alyona Lewis, Roger Chafe

**Affiliations:** 1 Division of Adolescent Health and Medicine, Department of Pediatrics, University of British Columbia, Vancouver, Canada; 2 Discipline of Pediatrics, Faculty of Medicine, Memorial University of Newfoundland, Room 409, Janeway Hostel, 300 Prince Phillip Drive, St. John’s, NL A1B 3V6 Canada; 3 Canadian Longitudinal Study On Aging (CLSA), Memorial University of Newfoundland, St. John’s, Canada

**Keywords:** Eating disorders, Young adults, Adolescents, Transition, User perspectives, Patient educational supports, Qualitative research, Thematic analysis

## Abstract

**Background:**

Eating disorders (EDs) commonly develop in adolescence and can be a chronic condition. Once patients reach the age when it is no longer permitted or appropriate for them to be seen in a children’s healthcare setting, they will need to transition into adult-focused care. This transition period can be challenging, with increased risks of negative health outcomes and disruptions in care. Appropriate educational resources could be an effective support for patients during this transition. Our objectives were to engage patients about the value of developing educational supports and determine how these supports should be structured to be most useful to young adults with EDs.

**Methods:**

Patients who had transitioned out of a hospital-based ED program between 2017 and 2020 were invited to participate in a semi-structured interview. Data were analyzed using thematic analysis and qualitative description.

**Results:**

Six young adults (5 females and 1 male) with EDs were interviewed. All participants thought it would be helpful to have an educational resource. Three main themes and seven subthemes were identified. Themes identified related to the unique challenges of transition for ED patients given the age of onset and cycle of symptoms; issues in adult care related to comorbidities and new level of autonomy; and the value of educational resources as both a connection tool and a benchmark. Participants also thought it would be useful to include in any educational resource a summary of their previous treatments, information regarding the transition process, a list of main healthcare providers they saw for their ED, a description of the differences and expectations of the adult system, a list of their follow up appointments, and a list of community and emergency mental health resources.

**Conclusions:**

Participants said that educational supports can play a useful role for young adults with EDs during their transition into adult care. They also provided valuable insights into the desired contents of such supports and expanded on the roles that educational resources could serve for ED patients.

## Abstract (summary)

Most adolescents who have an eating disorder will reach an age when it is no longer appropriate for them to receive care in a children’s health program. They will then need to transition to an adult-focused program. This transition period can be challenging, with increased risks of negative health outcomes and disruptions in care. One approach for better supporting patients during transition is through the development of appropriate educational resources. Before developing these resources, it is important to hear from patients about how they should be structured to be as useful as possible. We interviewed six patients who had recently transitioned out of a pediatric eating disorder program about the value of an educational transition resource and what should be included in it. Patients identified several unique transition issues for young adults with eating disorders. We identified valuable insights and seven key themes from these interviews. While all patients recognized the value of educational resources, rather than being just a static source of information, they envisioned a resource that could also be a dynamic record of their previous care and a tool for engaging with their new adult-focused health care providers.

## Background

Eating disorders (EDs), such as anorexia nervosa (AN) and bulimia nervosa (BN), are complex illnesses that are accompanied by serious medical, psychological, and social impairments [1]. They are associated with the highest rates of morbidity and mortality among mental illnesses diagnosed within the pediatric population [2]. Peak age of onset for AN and BN is middle to late adolescence and late adolescence (around 18 years) respectively [3, 4]. Because EDs can be chronic and have lasting functional and medical impairments, most ED patients will require transition to adult medicine once they have reached the age at which it is no longer appropriate or permitted for them to be seen in a children’s healthcare setting [5]. This transition typically occurs around age 18, however, an individualized process which considers the developmentally appropriate age for each patient to be transitioned is recommended [6].

The transition to adult care for patients with EDs has been associated with disengagement from services and inferior health outcomes [7]. Gaps in care for ED patients are also common between the pediatric and adult systems in many countries [7]. These gaps may contribute to the large declines in mental health service use and high treatment drop-out rates seen in this population after transition [8–11]. Dimitropoulos et al. found that common ED related factors, such as denial about their illness and ambivalence towards recovery, significantly affects this population’s transition to adult medicine [11, 12]. While pediatric care is more family-focused, adult care providers expect patients to take more responsibility for arranging and managing their own care [11, 12]. With this added autonomy, patients may be free to disengage from treatment if they do not feel it is warranted, resulting in difficult therapeutic relationships and disrupted care. Ensuring all aspects of continuity of care—e.g., maintaining care over time, ensuring a relationship between patient and care team, providing information transfer, and meeting changing needs [13]—during this period of establishing new relationships with adult-focused providers is a key goal.

While research surrounding the educational needs of patients with EDs during their transition is sparse, literature on the educational needs of patients with other chronic illnesses during transition may be applicable. A systematic review by Crowley et al. found that patient education is a vital component in optimizing health and empowering adolescents during transition [14]. They identified that patients should know about improved knowledge regarding their condition, the reasons for their current treatments, medication they are currently taking, the differences between the pediatric and adult healthcare systems, how to navigate the adult system, and personal wellness [15–17]. They also recommended that patients are provided with a comprehensive health summary prior to transferring them to the adult healthcare system [17, 18].

While there are likely areas of overlap, we should not just assume that the education needs of young adults with ED are like those of patients with any chronic disease. Within the health system we examined (in Newfoundland, Canada), adolescents with an ED are cared for in a medical program focused on treating all adolescent patients. Unlike other chronic illnesses in pediatrics, e.g., diabetes or cardiac care, general adolescent medical programs do not have a clear equivalent in adult medicine, which can complicate transitioning. Models of ED care likely vary in other countries or even between jurisdictions within a country. The treatment of EDs more regularly uses family-based therapies regardless of a patient’s age, in which parents take full control of a patient’s nutrition and slowly give responsibility back to the patient. This situation is different from other chronic illnesses, which often aim to empower patients relatively early to start taking control over their own care in preparation for the adult system. As a first step towards addressing gaps in the supports available, we engaged young adults with EDs to get their perspectives on the type and structure of educational resources that would be useful to them during their transition into adult care. Our objectives were to assess the value of developing educational supports tailored to this patient population and to determine how these supports should be structured specifically for young adults with EDs. The project was not driven by a theoretical perspective per se, but the need to address a practical problem of improving health care delivery for these patients [19]; and the belief that before developing resources to support transition, it is important to hear first from patients about how these resources should be structured to be as impactful as possible. While transition to adult care is a period of heighten risks, if properly supported, these transitions can also be great opportunities to prepare patients for their future adult lives.

## Methods

### Research design

This project was an applied qualitative research project, using both thematic analysis and qualitative description.

### Project team

The project team included (AD) a senior adolescent medicine pediatrician who had over 20 years of experience working with the ED population, (JM) a pediatric resident who was planning to pursue a career in adolescent medicine, and (RC) a health services researcher who has experience studying issues related to transitions to adult care and in qualitative research. One of the motivations for the project was that we all felt there were potential gaps in the transition programs for young adults with ED, potentially leading to disruptions in care and adverse outcomes for these patients. RC had recently completed a project with survivors of childhood cancer, where patients requested a locally relevant, age-appropriate educational resource to support their transition into adult care [20]. At the start of the project, the research team felt that a similar resource for patients with ED would likely be useful. The research team had some initial ideas around what content could be included in such a resource, following the structure of the resource developed and evaluated for the survivors of childhood cancer. We also felt that for the resource to be contextually relevant and as useful as possible to patients with EDs, that its content should be based on what they saw as their information needs and reflect their experiences with their transition, which motivated this project. A member of the research team (AD) would have been involved in the care of some of the study participants, but was not involved in the data collection for this project beyond identifying patients who met the study’s inclusion criteria.

### Setting and study participants

The Janeway Children’s Health and Rehabilitation Centre is the only tertiary care children’s hospital in the Canadian province of Newfoundland and Labrador (NL). The province has a population of approximately 520,000 people and covers an area greater than the size of Germany. The adolescent medicine program has responsibilities for treating cases of EDs across the province, with these patients being supported by a dedicated ED team. The inclusion criteria for this study were that a patient (1) had a previous DSM-5-TR consistent diagnosis of AN, atypical AN and/or BN; (2) had been followed by the Janeway hospital’s ED team for at least 1 year; and (3) were discharged into adult care between January 1st, 2017 and December 31st, 2020. There were no additional exclusion criteria.

Because of ethical considerations and concerns about sharing of personal health information, the administrative person for the Janeway’s ED team made initial contact with potential participants by telephone. After briefly explaining the study, this person asked for permission to give the patient’s current e-mail address to the study team. For those patients who allowed their e-mail address to be shared, a research assistant (AL) emailed participants a letter of invitation, which outlined the purpose of the study and the interview process; a copy of the patient consent; and the interview questions. For those still interested in participating in the study, a mutually convenient time was agreed to for an interview.

### Interviews

A semi-structured interview guide was developed by the research team reflecting the study objectives, based on similar interview guides previously used by members of the research team. The interview guide was piloted by clinical staff who had experience working with the study population to ensure comprehensibility and suitability before being used in the study. All participants were provided a written copy of the consent form prior to being interviewed. This form was reviewed by the interviewer before the start of the interview and an opportunity given to the participant to ask any questions. As explained to the participants, implied consent was deemed to be received by replying to the initial email sent by the research team and participating in the interview. All interviews were conducted by phone, lasting approximately 30 min in length. Interviews were all recorded and professionally transcribed. Transcripts were de-identified and assigned participants study code numbers.

The interviews covered the participants’ experiences transitioning into adult care, their views on how to improve that process, the potential value of educational supports and questions about the types of information that should be included in a patient educational resource. We used thematic analysis to analyze the interviews, following the approach proposed by Braun and Clarke [21, 22]. JM and RC both read all transcripts. RC coded all transcripts using both descriptive and in vivo codes [23]. JM and RC developed the initial themes. The research team then confirmed and finalized the main themes. For the sections of the interviews about the types of information that should be included in a patient educational resource, after completing the thematic analysis, we also analyzed those responses by providing a qualitative description [24, 25].

### Ethics

Ethics approval was obtained from the Newfoundland and Labrador Health Research Ethics Authority (Canada) [HREB # 2020.269].

## Results

A clerk for the adolescent medicine program identified twenty people who met the study inclusion criteria and who agreed to have their e-mail contact shared with the research team. The clerk then gave our team a list of these patients and their emails. All twenty were sent an e-mail invitation to participate in the study by our research team. Of these twenty, nine participants responded (8 females and 1 male) to our e-mail: eight accepting and one who declined our invitation to participate. The person who declined to participate left ED care as a youth and never experienced treatment as an adult and therefore deemed herself to be ineligible. Of the eight people who agreed to participate, two participants did not attend their interview and did not respond to subsequent requests to arrange an alternative time. Six participants (5 females and 1 male) completed interviews for the project.

Our analysis identified three main themes with seven subthemes (Table 1). Themes refer both directly to the issues related to transition process and to patients’ health status/needs during this period. The main themes related to the unique challenges faced by ED patients during their transition to adult care, the issues they encountered within adult care, and the value they saw in the development of an educational resource. Although these themes were not discussed in every interview, likely reflecting variation in patient experiences, none of the interviews conflicted with the themes presented below. Supporting quotes are provided from all six transcripts.

**Table 1:** Summary of Themes

Theme	Subthemes
Theme 1. Unique challenges for ED patients	Subtheme 1: Late age of onset Subtheme 2: Cycle of symptoms Subtheme 3: Transition versus discharged
Theme 2: Issues in adult care	Subtheme 1: New autonomy Subtheme 2: Comorbidities
Theme 3: The value and content of educational resource	Subtheme 1: Resource as a connection tool Subtheme 2: Resource as a benchmark

### Theme 1: unique challenges for ED patients

All participants found at least parts of their transition to adult medicine difficult. Some of the issues discussed are similar to issues reported by patients with other chronic conditions, e.g., not understanding the transition process or not being familiar with the adult healthcare system. Those who transitioned during the COVID pandemic said that this period caused additional challenges, particularly related to long wait times for adult services. Other challenges that participants identified were unique to the ED population.

### Subtheme 1: late age of onset

“I started the pediatrics program when I was 17, I got referred there. About a year after, I got transferred to the adult system.” [Participant 3]

In comparison to other medical conditions seen within pediatrics, the relatively late age of onset for ED means that the patient may not have a long relationship with their care team.

There are potentially both advantages and disadvantages to this situation. For other chronic diseases, the fact that families and patients have built, and now have to break, their long relationship with their child’s healthcare providers has been reported as an added burden during transition [26]. The lack of a long therapeutic relationship may lessen the emotional burden of some ED patients as they leave pediatric care. On the other hand, depending on the patient, because of this relatively short therapeutic relationship, they could be left without feeling a strong connection to either their pediatric or adult care team. This situation could lead to adverse outcomes if the patient needs added support during their transition. Another factor is the intensity of the caring relationship. Some patients reported having extensive periods of in-patient hospital care, which could help them form a close connection with their providers over a short period. Finally, this relatively short period of treatment may result in suboptimal weight restoration, symptom control, and cognitive rehabilitation required to fully comprehend and retain education about eating disorders, long-term impacts, and the recovery process. It is unclear how this relatively short duration of illness before transition impacts a patient’s knowledge of and ability to self-manage their condition, but may lead to increased educational needs during this period.

### Subtheme 2: cycle of symptoms

“Comparable to a wave. So sometimes they [the symptoms] were stable and sometimes they were up and down.” [Participant 2]

Recovery is not a linear process. It is common for ED patients to go through cycles during which their symptoms worsen or improve. This fluctuating road to recovery may impact their transition experience, particularly if they are doing well at their time of transition and did not require close follow up. Four of the six participants said that the severity of their symptoms changed in the period after leaving pediatric care, a time when they may not have had specific ED follow up in place yet and were trying to navigate a new healthcare system. The transition experience itself can also negatively impact patients’ symptoms due to the change in independence, as expressed by Participant 5.

“After you’ve been discharged, there’s a point, where I guess, you don’t really receive direct care anymore… and there’s not really anyone watching you as closely, kind of thing. So, I would definitely say the symptoms worsened.” [Participant 5]

### Subtheme 3: transition versus discharged

“When I was discharged from the Janeway, I think one of the hardest things after that… so once I went home, I had my appointments that I was supposed to go to and stuff, but I still felt like a bit of disconnect, if that makes sense. I kinda didn’t really know what to do and what I was able to do and I don’t know… just mentally, it was very challenging.” [Participant 5]

The patients interviewed all discussed being hospital inpatients as part of their ED treatment. The discharge from an in-patient hospital-based ED program can be quite dramatic for patients, possibly even more so than their transition into adult care. After leaving hospital, for those under 18, they would return home but continue to be regularly seen on an outpatient basis by the ED team. Because ED patients are seen in Newfoundland through the Adolescent medicine program, there is no clear corresponding program on the adult side, as there is for example between pediatric and adult endocrinology. At the age of 18, depending on the status of the patient, they would have most likely been referred to a community-based eating disorder program or discharged from care. Within the context of the late age of onset and the cycle of symptoms, patients interchanged discussions of being discharged and transitioned. Other participants contrasted transitioning with discharge.

“The process was very abrupt.… I basically had an appointment, and she [the healthcare provider] said… ‘You’re discharged.’ And I was like, oh okay. Sounds good. And that was pretty much the discharge process. I wasn’t transitioned over.” [Participant 6]

Overall, it is important for patients to understand that discharge does not mean recovery. If there are signs of relapse, patients should know where to reach out for care and be encouraged to maintain regular medical follow up with their family physician.

### Theme 2: issues in adult care

Unique issues related to EDs also impacted the participants’ experiences of adult care. These issues included their current understanding of adult care and the impacts brought about by the change in treatment care models.

### Subtheme 1: new autonomy

“I know from personal experience, there is a difference in how they treat you. In regards to when you’re an adult with an eating disorder, it’s treated as it’s your responsibility and it’s your choice, however, that’s not necessarily the case. Whereas in the Janeway, they get families involved to help with the program, so there are some differences.” [Participant 3]

A significant change between pediatric and adult care is that in the adult system, the patient is expected to take responsibility for managing their own care after transition, whereas in pediatric care, family-based therapy is the mainstay of treatment, which involves the caregivers taking over complete responsibility of their child’s nutrition, ED symptoms, and medical follow up for some amount of time. Without proper education, this new autonomy can be an unexpected change: “it was surprising to me that I had so much choice in the matter of my treatment” [Participant 4]. For a condition like ED for which some patients may choose not to seek treatment due to ambivalence about recovery or denial about their illness, this new autonomy can be a significant issue. As Participant 1 said, “I felt really confident during my treatment when I was an adolescent, however… I found it was a lot more self assessment as I got older, which was harder for me.”

In the above quote from participant 3, there is also the sense that adult providers inadvertently imply that the ED and recovery process is the patient’s choice. This perspective and way of communicating would be significantly different from that taken by pediatric providers, particularly within the context of an ED program. If adult providers did imply the person’s ED as their choice, this view could put added pressure on the therapeutic relationship. It also points to the need for some education directed towards adult providers who may support patients with ED but who may not have a deep understanding of the ED population and how to communicate with them appropriately.

### Subtheme 2: co-morbidities

“I think regardless if you’ve had an eating disorder, if you’ve been admitted to the hospital or whatever they do, because this is obviously a mental health disorder” [Participant 2].

Some participants classified ED as a mental health disorder. Other participants said that mental health conditions are common comorbidities for patients with an ED. Participants expressed the view that the adult resources available to them through community programs only focused on the patient’s ED symptoms, while not addressing any co-morbidities. As Participant 3 said: “the primary focus in an ED program is an ED, but they shouldn’t just deny or sum it all to one illness, when there are other illnesses at stake.” Even if it did not impact the patient’s ED symptoms, participants said that transition and the period of being a young adult can still be difficult on patients’ mental health.

“I believe that my [ED] symptoms remained pretty stable during the transition…. I think the more generalized mental health got worse. You know, things that come with EDs, other symptoms like depression and anxiety, things like that, got a little bit worse.” [Participant 4]

### Theme 3: the value and content of educational resource

All the participants said that it would be “definitely” or “absolutely” helpful to have an educational resource to better support transition. Some were surprised that such a resource does not already exist. Participants also discussed the potential of an educational resource beyond it being just a static source of information directed towards young adult patients.

### Subtheme 1: resource as a connection tool

“I found it a little bit difficult because I kinda felt like I didn’t know where all my information was and it was harder to get all that information from the Janeway and bring it to the Adult care… it would give the Adult team a little bit more understanding of what you had previously gone through and what treatment you had received. Because it would help them understand… what has worked for you and what has not worked for you.” [Participant 4]

When the research team initially considered the educational resource, we saw it as a static way to present information to young adults with ED as a means of better informing them about their condition and their move to adult care. A common theme was that participants saw the value of this type of education resource as a way of better engaging with providers on the adult side. Participants envisioned the resource having a summary of their previous interactions with the pediatric system, e.g., drugs prescribed, providers seen. While they still wanted standard information about ED included, patients also wanted this resource to be a kind of personal health passport which they could share with their new providers. “It would be helpful for the new professionals to understand you better” [Participant 3]. Some of this information would be in the patient’s electronic health record, but it is not clear which providers could access the patient’s records, particularly if post-transition treatment is received through a community-based program. Having this information in a resource that the patient has would also allow them to be more engaged in discussions about their past care and future directions.

### Subtheme 2: resource as a benchmark

“I had a bunch of tests done when I first got admitted to the Janeway and I honestly would like to see where my body is now compared to where it was then. Yes, I think it would be very helpful.” [Participant 2]

Participants saw value in having a history of their medical record, including professionals they saw, diagnostic imaging reports and bloodwork results, as a way of benchmarking where they are with their condition. As mentioned above, one of the features of having an ED is that the symptoms often go in cycles. Some patients also make significant progress in treating their condition. Being able to clearly see the improvements in their physical state may help both with the appreciation of the progress and as a motivator for maintaining their positive health gains. For patients who see signs of deterioration in some health measures, providers could discuss ways that patients can return to their previous state of health. Of note, some patients’ distorted ED thoughts applaud them for being ‘sicker’ therefore, this should be a consideration when sharing results with the patient.

#### Contents of the educational resource

We asked participants about the type of content that they thought should be included in any educational resource. Specifically, we ask whether patients thought it would be useful to include either a summary of their previous treatments, information regarding the transition process, a list of main healthcare providers they saw for their ED, a list of previous and current medications, results of tests completed before transition, a description of the differences and expectations of the adult system, a list of follow up appointments and a list of community and emergency mental health resources. Overall, participants felt that all this information would be useful to include in an educational resource. The benefit that patients saw about including this information mostly related to the benefits for engaging with new providers and benchmarking their physical state versus their performance on previous tests.

## Discussion

Transition to adult care can be a difficult period for young adults. Our analysis identified unique challenges faced by ED patients, including those related to the relatively late age of onset, the cycle of symptoms, the treatment of comorbidities after transition, and the new level of autonomy. Participants in this study all felt that educational supports can play a useful role for young adults with EDs. Developing and evaluating such educational resources would be a productive step towards better supporting ED patients as they move into adult care and potentially prevent medical and psychological deterioration that can accompany this transition. At the start of the project, the research team felt that such an educational resource was needed. This view was confirmed during the study. Our analysis did change however our perspective of the function that educational resources could play for ED patients and what should be included in them.

Some of the information identified in the analysis would be relatively straightforward to include in an educational resource, e.g., clarifying the distinctions between discharge, transfer and transition. The need for more information about the adult system and a description of new expectations/autonomy that patients are likely going to face could also be added. Participants envisioned an educational resource that would include patient specific information, including a comprehensive history of the medical treatments received (e.g., inpatient treatment, outpatient treatment, medications trialed, counselling, etc.) and important investigations completed (e.g., Bone scan, echocardiogram, electrocardiograms, brain MRI, hormone testing, electrolytes). We would not include specifics around their weights or meal plans due to possible triggering information and would need to check with providers about the viability of providing a detailed account of a patient’s medical history given the differing responses patients with EDs may have to medical progress. Participants saw this information as something that they could review themselves as a benchmark and as something that they could share with adult providers to help them to direct future care. While the information is similar, these are two different roles and it would have to be determined how best to serve each function. Participants also suggested giving links or phone numbers to adult providers and community-based mental health supports to access when in crisis. While this information could be provided, it may require that the resource is able to be updated to ensure that any links remain active, which could impact considerations of whether it is a paper-based or electronic resource.

Once we develop an educational support, it is important to consider the timing for introducing it to patients, especially given the late age of onset that some patients with EDs have in relation to the age limits of pediatric care. Planning a patient’s transition should occur long before it happens [27]. Any transition supports can be introduced to patients as soon as the care team are comfortable that the patient can safely discuss post-pediatric care. Staff can work through the resource during a patient’s time in the pediatric system and continue to update key learnings. As it is reviewed closer to transition, the resource could serve as a prompt to review education around the adult system, the patient’s goals, available resources, and to whom the patient will be transitioning. After the transition occurs, the patient can have the tool as a health summary for new providers and a resource that contains information about their ED, mental health supports, and emergency contacts. The tool can also be reviewed by adult providers to help ensure that unnecessary gaps in care or in the information needs of young adult patients do not occur.

The project had a number of strengths. It is one of the first to engage ED patients about their educational needs during transition. Our project team blended both clinical and research expertise and had a sharp applied focus on the patients’ educational needs during the transition period. We identified factors related to the transition of ED patients not previously identified in the academic literature. The project also had several limitations. This study focused on a single program within one Canadian province. The results may not be generalizable to other programs or settings. Transition-aged patients can be difficult to recruit in research studies [28]. Our recruitment may have been impacted by the ED team having to first contact patients before they could be contacted by the research team as we were directed to do by the research ethics board. Other factors which may have impacted participation included that recruitment occurred during the COVID-19 pandemic, participants had already transitioned and were no longer seen by the ED team, the fact that these patients are now adults and may no longer reside in the same province nor have the same contact information as they did when being treated by the ED program. When specifically asked about information that would be beneficial to include, most participants agreed to all presented options. Instead of directly asking about the value of including specific types of information, we could have asked participants without prompts or we could have asked participants to rank their preferences for including various types of information. Next to the gender of the participants and confirming that they met the inclusion criteria prior to their interview, we did not collect any further demographic information which limited our ability to conduct any sub-population analysis within our study. Participants were able to discuss in their interviews how their backgrounds and experiences impacted what they felt should be included in an educational resource.

## Conclusions

Transition can be a difficult period for patients, particularly for young adults with eating disorders. The development of appropriate educational resources is one approach for better supporting patients. Before developing such resources, we found it useful to engage patients who could be potential users to gain insights about how such a resource should be structured and what material should be included in it. Some of our findings, e.g., those related to the unique themes patients with eating disorders face during transition, are likely true for patients in other programs. Some of the other findings may be more reflective of patients within the particular program we examined. We would recommend that other eating disorder programs look at developing educational resources to support their patients during transition and that there is likely value in engaging patients in their own local contexts about how such a resource could be structured to best meet the needs of their patients.

Our next step is to develop and evaluate a transition resource. We greatly value the insights that the patients gave. In developing this resource, we will need to investigate what information, particularly around the care of individual patients, can be included. There are several issues that need to be considered, e.g., how to present information in a meaningful and constructive way for patients; that the level of information is not too resource intensive for the pediatric program to provide; and, if the resource is going to be used as a tool for engaging providers on the adult side, what information would be useful to provide to them. With that said, it is our intension to follow as closely as we can the guidance given to us by the patients who participated in this study. Transition can be difficult, but by working together and listening to the directions given to us by patients who have recently transitioned, we will be able to develop a more appropriate resource and improve the experience for future patients.

## Data Availability

The interview data are not publicly available, although de-identified transcript data could potentially be made available. Please send requests to access the data to the corresponding author.

## Abbreviations

AN: Anorexia nervosa
BN: Bulimia nervosa
DSM-5-TR: Diagnostic and statistical manual of mental disorders, text revision, fifth edition
ED: Eating disorders
